# Analysis of medication management system data to determine potentially inappropriate medication use and hospitalization among older adults living in residential care homes for the elderly population

**DOI:** 10.1186/s12877-025-05989-4

**Published:** 2025-05-06

**Authors:** Ho Cheung Chau, Kexin Zhang, Bik-Wai Bilvick Tai, Isaac Shing Yan Hui, Hon Ming Ma, Martin Chi Sang Wong, Sau Chu Chiang, Yin Ting Cheung

**Affiliations:** 1 Hong Kong Pharmaceutical Care Foundation Limited, Room 703-704, 7th Floor, CRE Center, 889 Cheung Sha Wan Road, Kowloon, Hong Kong SAR China; 2 School of Public Health, Fudan University, Shanghai, China; 3 School of Pharmacy, Faculty of Medicine, The Chinese University of Hong Kong, 8th Floor, Lo Kwee-Seong Integrated Biomedical Sciences Building, Area 39 Shatin, N.T, Hong Kong SAR, China; 4 Faculty of Science, University of Bath, Bath, UK; 5 JC School of Public Health and Primary Care, Faculty of Medicine, The Chinese University of Hong Kong, Hong Kong SAR, China

**Keywords:** Older adults, Nursing home, Residential care homes, Potentially inappropriate medication, Polypharmacy, Beers Criteria, Pharmacists, Medication review

## Abstract

**Objectives:**

Many older adults living in Resident Care Homes for the Elderly (RCHEs) are at risk of polypharmacy and the use of potentially inappropriate medication (PIM). Few studies have evaluated the prevalence and consequences of PIM use among older adults living in RCHEs. The objectives of this study are (1) to evaluate the prevalence of PIM use in 29 RCHEs in Hong Kong, and (2) to investigate the association between PIM use and hospitalization in this population.

**Methods:**

This is a prospective, observational, cohort study which utilized final-administered medication data from RCHEs that participated in a medication management program. Data on the medications administered to all residents living in the participating RCHEs were extracted from the SafeMed Medication Management System (SMMS^®^), which is a purpose-built Information Technology supporting the entire medication management process at RCHEs. The outcome of interest is the 12-month period prevalence of PIM use (January 1 to December 31, 2023), which was obtained by comparing the medication data with the 2023 Beers criteria. Hospital admissions during the study period were extracted from the SMMS^®^.Multivariable logistic regression was conducted to investigate the association between PIM use and hospital admissions.

**Results:**

We included 6,346 residents (age 82.9 ± 8.6 years; female 61.9%). The average number of current medications was 6.8 ± 7.4. Over half (51.5%) of residents had polypharmacy (≥ 5 medications). The 12-month period prevalence of PIM use was 34.5%. Among the residents with PIMs, 65.1%, 25.5% and 9.4% used 1, 2 and > 2 PIMs, respectively. Residents with PIMs were associated with higher rates of hospitalization (Odds Ratio [OR] 1.73, 95% confidence interval [CI] 1.54 to 1.69), after adjusting for age, sex and comorbidities. The number of PIMs was significantly associated with higher risk of hospitalization (OR: 2.17, 95% CI: 1.82 to 2.59 for > 1 PIMs vs. 0).

**Conclusions:**

The use of PIM was observed in one-third of older adults living in RCHEs, and was associated with an increased risk of hospitalization. Our findings highlighted the urgent need for strategies to improve clinicians’ awareness of PIMs and their adverse impact, and to implement pharmacist-led medication reviews in RCHEs.

**Supplementary Information:**

The online version contains supplementary material available at 10.1186/s12877-025-05989-4.

## Introduction

Physiological changes associated with aging can alter the pharmacokinetics and pharmacodynamic properties of drugs. These factors play a significant role in augmenting drug toxicity in adults aged 65 or over (hereinafter, “older adults”), increasing their susceptibility to adverse drug reactions (ADRs) associated with the use of multiple drugs [1, 2]. Moreover, older adults may take potentially inappropriate medications (PIMs), which are “*drugs with… risks [that] outweigh potential benefits*,* especially when effective alternatives are available.*” [3]. To reduce older adults’ exposure to PIMs, the American Geriatrics Society (AGS) developed the Beers Criteria for PIM Use in Older Adults as a safe-prescribing reference for clinicians [4]. Although the Beers criteria were designed for use in the US, they have been widely used to assess the prevalence of PIM use by older populations in various settings in other countries, including Hong Kong [5, 6, 7, 8].

Many older adults living in residential care homes for the elderly (RCHEs) have multiple chronic diseases and thus have a higher probability of having polypharmacy (i.e. receiving five or more medications concurrently) [9, 10]. For example, it has been reported that over 10% of the Slovenian population had polypharmacy, and 4% with over ten medications concomitantly (i.e., with hyper-polypharmacy) [11]. A systematic review of 17 studies found that according to the Beers criteria, the prevalence of PIM use by RCHE residents ranged from 18.5–82.6%.^12^ It has been shown that compared with appropriate medication use, inappropriate medication and PIM use by older adults is associated with more frequent hospital visits and hospitalization [12, 13]. In particular, inappropriate use of medications in older adults with psychiatric conditions may lead to increased risk of developing major cardiopulmonary and neurological adverse drug reactions [14]. Furthermore, a Korean study reported that residents who used ≥ 4 PIMs (according to the Beers and Korean criteria) at the time of RCHE admission had a 30% increased risk of an emergency department visit [15].

In Hong Kong, older adults comprised 20.5% of the population in 2021, and this percentage is expected to increase to 36.0% of the population by 2046 [16]. The 2021 Hong Kong Population Census reported that 95.2% of older adults were living in domestic households, while the remaining 4.8% were living in non-domestic households, such as RCHEs [17]. Thus, improving medication safety and management has become a priority of the Hong Kong healthcare agenda [18]. An analysis of data obtained from the Hospital Authority, a statutory body that governs all public healthcare services in Hong Kong, reported that the 12-month prevalence of PIM use was 55.5% in 2006 and reduced to 47.5% in 2014 [19]. However, there remains a lack of information regarding the risk of hospitalization due to the use of PIMs in older adults living in RCHEs in metropolitan cities like Hong Kong. Furthermore, the accuracy of the medication exposure data in this study was limited, as these data were obtained from a repository that captured information on dispensed medications rather than administered medications.

### Study objectives

The objectives of this study are (1) to describe the prevalence of PIM use according to the 2023 Beers criteria in older adults living in RCHEs of Hong Kong, and (2) to investigate the association between their PIM use and risk of hospitalization.

## Methods

### Study design

This is a prospective, observational, cohort study that analyzed data collected from the Integrated Old Age Home Medication Management Program, which was initiated by the Hong Kong Pharmaceutical Care Foundation Ltd (HKPCF), a non-governmental organization. The medication management program employs various technologies and automated methods to develop a medication management system for participating RCHEs in Hong Kong [20].

Approval was obtained from the Survey and Behavioral Research Ethics (SBRE) Committee of the Chinese University of Hong Kong (reference number: SBRE-19-106). All participating RCHEs provided consent for the use of de-identified data for research purposes. The implementation and use of the SMMS^®^ at the participating RCHEs is funded by a philanthropic organization, which played no role in investigating, analyzing or interpreting the results of this study.

### Base cohort

This study analyzed medication and clinical data of the residents living in 29 RCHEs that participated in the medication management program from January 1 to December 31, 2023 (the study period) across various geographical regions of Hong Kong. These RCHEs met the SMMS^®^ criteria, i.e., they (1) had a Wi-fi network, (2) received sustained operational funding from the government, the private sector, or philanthropic organizations, and (3) consented in writing to provide de-identified data for medication management program evaluation and research purposes. To ensure generalizability to older adults living in RCHEs of Hong Kong, the data of all residents who were aged 65 years or older and living in the participating RCHEs (*n* = 6,346) was analyzed in this study, regardless of their medication status (Supplemental Fig. 1).

### Data source

All data of the base cohort were extracted from the SafeMed Medication Management System (SMMS^®^), which is an electronic medication system developed by HKPCF with diverse applications for RCHEs to integrate patients’ medication data from different sources. In Hong Kong, RCHE residents are prescribed medications by physicians at hospitals or outpatient clinics (i.e. General Out-patient Clinic [GOPC]) under the Hospital Authority, which is a public health system funded by the Government and is responsible for more than 90% of the in-patient service in Hong Kong. To a lesser extent, general practitioners and clinicians from private clinics or hospitals may also prescribe medications for patients. The medications were collected by RCHE staff, who also entered patients’ information and medications into the database of the SMMS^®^ [20].

Unique among systems for RCHEs in Hong Kong, the SMMS has included a comprehensive drug database that covers all medications commonly prescribed by clinicians from the Hospital Authority. The database includes the details of each medication as follows: trade name, dosage form, strength, legal classification, therapeutic class (according to the Anatomical Therapeutic Chemical Classification [21]), precautions, common instructions for use, Hong Kong registration number, and manufacturer. This drug database enables the systematic built up of medication profiles for individual residents with detailed appropriate drug administration schedules. Moreover, the system supports electronic records of medications that are administered to the residents of RCHEs at the scheduled times in a real-time manner. Thus, the administered medication data for the base cohort were extracted from the backend of the SMMS^®^ database. The SMMS^®^ database also contains updated information on RCHE residents’ personal information, and follow-up consultations at hospitals or clinics, hospital admissions and discharges. These data are reliable and comprehensive, as the SMMS^®^ is the sole medication management system for all 29 participating RCHEs. Our previous study determined that the amount of data missing from the SMMS^®^ database is negligible [20]. For major comorbidities and medical conditions, the nursing staff of RCHEs manually entered the residents’ existing health conditions from the medical records of the Hospital Authority (including hospitals and outpatient clinics) upon initiation of a profile for each new resident, and updated the list periodically based on the latest health status of the resident. For the purpose of the current analysis, coding of the health conditions was conducted by one investigator (ISYH) and independently verified by two other investigators (KZ and YTC). In addition, all records in the SMMS^®^ database are anonymized and de-identified for research purposes.

### Operational definition of PIM use

The 12-month prevalence of PIM use was calculated by dividing the number of members of the base cohort with at least one PIM use from January 1 to December 31, 2023 by the total number of residents in the 29 RCHEs during the study period.

The 2023 AGS Beers criteria contain five categories of PIM use, [4] of which four of these were excluded from the current study. The updated Beers criteria involved an expert panel of highly experienced specialists with a robust internal review and external public comment processes [4]. Specifically, the categories of “medications potentially inappropriate in patients with certain diseases or syndromes” and “potentially inappropriate drug-drug interactions” were excluded due to concerns regarding their lack of comprehensiveness, as disclaimed by the developers, [4] and because diagnosis information and the exact indications of medications are not available in the SMMS^®^ database. In addition, the category of “medications to be used with caution” was excluded, as such medications can be used under specific circumstances. Moreover, the category of “medication with dosage adjustments based on renal function” was excluded, as assessment of kidney function requires clinical data that are not available in the SMMS^®^ database. Therefore, the only category included in this analysis was “medications considered potentially inappropriate independent of diagnosis.”

Subsequently, the drugs in this category were examined in the context of their registration status in Hong Kong [22]. PIMs that are not commercially registered in Hong Kong (such as meclizine, amoxapine, and desipramine) were excluded. In addition, PIMs with inappropriateness defined by their indication, dose, or therapy duration were excluded, because the SMMS^®^ database does not capture the aforementioned data. Thus, the final PIM assessment criteria were adapted to Hong Kong and contained 36 PIMs independent of diagnosis (Supplemental Table 1). This approach of defining PIMs and the 36 PIMs in this analysis are consistent with previous studies that have investigated drug utilization among older adults in Hong Kong [19, 23].

### Hospitalization data

To examine the association between PIM use and hospitalization rates, another cohort was constructed from the base cohort, consisting of residents ≥ 65 years old in the 29 RCHEs who were administered at least one medication between January 1 and December 31, 2023 (Supplemental Fig. 1). Residents not taking any medications were excluded from this analysis as they might be in better health state and had fewer severe comorbidities; including these healthier individuals could cloud the observed rate of hospitalization in non-PIM users, potentially leading to biased results. From this sub-cohort, information on hospitalization between January 1 and December 31, 2023, comprising the dates of admission and discharge, as well as the type of care received were extracted from the SMMS^®^. Only hospital admission(s) that occurred after the start date of any PIM and before the end date (if any) for each resident was coded as a “case” in this analysis. All types of hospitalization, regardless of planned or unplanned admissions and length of stay, were included.

### Statistical analysis

Descriptive statistics were used to summarize the characteristics of the base cohort and medications they were administered.

The association between the use of PIM and hospitalization in the base cohort was evaluated using multivariable logistic regression. The main predictors of interest were the use of any PIMs (yes vs. no) and the number of PIMs used (0 or 1 vs. ≥ 2) and were analyzed in separate models to prevent multicollinearity. The other covariates included in the model were clinically relevant factors that have been reported to be associated with hospitalization in institutionalized older adults, [24, 25, 26] namely sex, age (65–74, 75–84, or 85–94 vs. ≥ 95 years), number of concurrent medications (0–4 or 5–9 vs. ≥ 10), and number of comorbidities (0–2, 3–5, or 6–8 vs. ≥ 9). The magnitude of associations was quantified by calculating the odds ratios (ORs) and 95% confidence intervals (CIs). A P value of less than 0.05 was considered to indicate a statistically significant difference. R V.4.4.0 software was used for all statistical analyses.

As our study did not capture the actual ADRs experienced by the study population, an exploratory descriptive analysis was conducted to estimate the cumulative burden of ADRs due to PIMs in each member of the base cohort. The estimated ADR burden was quantified using the Cumulative Toxicity Tool in Polypharmacy Guidance Realistic Prescribing 2018, which cross-tabulates 32 classes of medication (including PIMs) and 15 ADR risks [27]. A risk score was used to quantify the cumulative risk of each ADR. Specifically, members of the base cohort who took a drug associated with an ADR were assigned a risk score of 1, and if they took multiple drugs associated with the ADR, the corresponding risk scores were summed to obtain a total risk score. A higher risk score indicated a high risk of experiencing a given ADR and represented the cumulative burden of the ADR in a given member of the base cohort.

## Results

### Characteristics of the base cohort

The mean age of the base cohort was 82.9 (standard deviation (SD) = 8.6) years, and 61.9% were women (Table 1). The average number of concurrent medications was 6.8 (SD = 7.4), and polypharmacy (i.e. receiving five or more medications concurrently) was present in approximately 51.5% (*n* = 3,266) of the base cohort using medications most often for comorbid hypertension (*n* = 3,290, 51.8%), dementia (*n* = 2,201, 34.7%), or acute infection (*n* = 2,095, 33.0%). The prevalence of the 32 comorbidities in the base cohort are shown in Supplemental Table 2.

The medications used in the 12-month study period by the base cohort are shown in Supplemental Table 3. The most commonly used medications were those that act on the gastrointestinal system (*n* = 4,381, 69.0%), central nervous system (CNS; *n* = 4,350, 68.5%), and cardiovascular system (*n* = 4,081, 64.3%). The three most commonly used medications that act on the gastrointestinal system were bisacodyl (*n* = 2,999, 47.3%), senna (*n* = 2,811, 44.3%), and lactulose (*n* = 2,595, 40.9%).

**Table 1:** Characteristics of the base cohort (*N* = 6,346)

Characteristics	*n* (%)
**Sex**	
Female	3,931 (61.9)
Male	2,415 (38.1)
**Age (years), mean ± SD**	82.9 ± 8.6
65–74	860 (13.6)
75–84	1,649 (26.0)
85–94	2,893 (45.6)
≥ 95	944 (14.9)
**Major comorbidities**	
Hypertension	3,290 (51.8)
Dementia	2,201 (34.7)
Acute infection	2,095 (33.0)
Stroke	1,985 (31.3)
Eye disease	1,799 (28.3)
Renal disease	1,671 (26.3)
Diabetes	1,648 (26.0)
Cholesterol	1,431 (22.5)
Fractures	1,336 (21.1)
Psychiatric	1,051 (16.6)
Arthritis	989 (15.6)
Cancer	727 (11.5)
Anemia	682 (10.7)
Ischemic heart disease	659 (10.4)
**No. of concurrent medications, (mean ± SD)**	6.8 ± 7.4
0	2,786 (43.9)
1–4	294 (4.6)
5–9	879 (13.9)
10–14	1,273 (20.1)
≥ 15	1,114 (17.6)
**No. of classes of concurrent medications, (mean ± SD)**	5.2 ± 6.3
0	2,786 (43.9)
1–5	527 (8.3)
6–10	1,354 (21.3)
11–15	1,193 (18.8)
≥ 16	486 (7.7)
Prescribing sources of medications*	
Public hospitals only	3062 (86.0)
Private hospitals only	147 (4.1)
Public and private hospitals	351 (9.9)

SD: standard deviation

*Include only 3,560 residents who were prescribed at least 1 medication

### Prevalence of PIM use

At least one PIM was taken by one-third (*n* = 2,189, 34.5%) of the base cohort, with 65.1% (*n* = 1,425) taking one PIM, 25.5% (*n* = 558) taking two PIMs, and 9.4% (*n* = 206) taking more than two PIMs. Table 2 presents the PIMs that were taken by over 1% of the base cohort. The most frequently used PIMs were chlorpheniramine (*n* = 1,031, 16.2%), lorazepam (*n* = 382, 6.0%), promethazine (*n* = 288, 4.5%), and gliclazide (*n* = 280, 4.4%). A full list of the prevalence of PIM use is presented in Supplemental Table 4.

**Table 2:** Top 10 most frequently administered potentially inappropriate medications in the base cohort (*N* = 6,346)

Medication	Therapeutic category	No. of users	Prevalence of PIM use (%)
**Chlorpheniramine**	First-generation antihistamines	1,031	16.2
**Lorazepam**	Benzodiazepines	382	6.0
**Promethazine**	First-generation antihistamines	288	4.5
**Gliclazide**	Sulphonylureas	280	4.4
**Clonazepam**	Benzodiazepines	175	2.8
**Diphenhydramine**	First-generation antihistamines	171	2.7
**Trihexyphenidyl**	Antiparkinsonian agents	125	2.0
**Hydroxyzine**	First-generation antihistamines	109	1.7
**Zolpidem**	Nonbenzodiazepine	105	1.7
**Scopolamine (Hyoscine)**	Gastrointestinal antispasmodics	102	1.6
**Cyproheptadine**	First-generation antihistamines	81	1.3

### Association between PIM use and hospitalization

In the study period, 4,686 members of the base cohort were administered at least one medication, and 2,563 (54.6%) were also hospitalized. The characteristics of those who were and were not hospitalized are presented in Supplement Table 5.

In the multivariable model (Table 3), compared with those who did not use PIMs, those who did use PIMs had higher rates of hospitalization (OR: 1.73, 95% CI: 1.54 to 1.69), and the number of PIMs they used was positively associated with their risk of hospitalization (OR: 2.17, 95% CI: 1.82 to 2.59 for > 1 PIMs vs. 0). Those with a high comorbidity burden had a greater risk of hospitalization than those without comorbidity (OR: 1.72, 95% CI: 1.45 to 2.06 for ≥ nine comorbidities vs. one comorbidity). Compared with those who received zero to four medications, those who received five to nine medications had lower risk of hospitalization (OR: 0.72, 95% CI: 0.60 to 0.86) while those who received ten or more medications was not associated with hospitalization (OR: 1.00, 95% CI 0.87 to 1.14).

**Table 3:** Factors associated with hospitalization risk

	Model 1		Model 2	
Variables	**OR (95% CI)**	***P***	**OR (95% CI)**	***P***
**PIM use in 2023**				
No	Reference		---	
Yes	1.73 (1.54–1.96)	**<0.001**	---	
**No. of PIMs in 2023**				
0	---		Reference	
1	---		1.56 (1.36–1.78)	**<0.001**
≥ 2	---		2.17 (1.82–2.59)	**<0.001**
**Sex**				
Male	Reference		Reference	
Female	0.79 (0.69–0.90)	**<0.001**	0.79 (0.69–0.90)	**<0.001**
**Age**				
65–74	Reference		Reference	
75–84	0.93 (0.76–1.14)	0.50	0.94 (0.77–1.16)	0.5860
85–94	1.22 (1.00-1.47)	**0.046**	1.23 (1.02–1.49)	**0.032**
≥ 95	1.41 (1.12–1.77)	**0.003**	1.44 (1.15–1.82)	**0.002**
**No. of concurrent medications**				
0–4	Reference		Reference	
5–9	0.72 (0.60–0.86)	**<0.001**	0.72 (0.61–0.86)	**<0.001**
≥ 10	1.00 (0.87–1.14)	0.98	0.99 (0.86–1.13)	0.83
**No. of comorbidities**				
0–2	Reference		Reference	
3–5	1.16 (0.99–1.36)	0.063	1.16 (0.99–1.36)	0.065
6–8	1.33 (1.13–1.57)	**<0.001**	1.32 (1.12–1.56)	**<0.001**
≥ 9	1.72 (1.45–2.06)	**<0.001**	1.71 (1.43–2.04)	**<0.001**

OR: odds ratio; PIM: potentially inappropriate medication; 95% CI: 95% confidence interval

Model 1: Multivariable logistic regression model with PIM use, sex, age, number of concurrent medications and number of comorbidities as independent variables

Model 2: Multivariable logistic regression model with number of PIMs, sex, age, number of concurrent medications and number of comorbidities as independent variables

### Exploratory analysis: estimating the cumulative burden of ADRs in PIMs

**Fig. 1:**
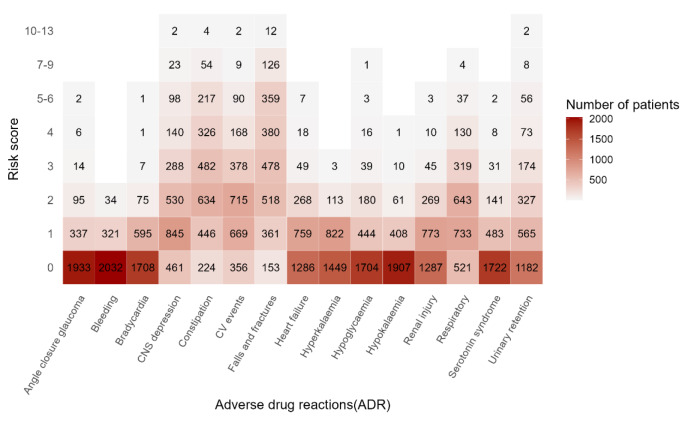
*The risk scores for 15 distinct adverse drug reactions (ADR) among 2,387 residents with hyper-polypharmacy (i.e. taking ≥ 10 medications) *The x-axis represents the 15 types of adverse drug reactions as outlined by the Cumulative Toxicity Tool of the Polypharmacy Guidance 2018, while the y-axis represents the cumulative risk scores. Each square denotes the specific risk score, and the number within each square indicates the number of patients at that risk score level. A darker color indicates a higher number of residents bearing that risk score level CNS depression: central nervous system depression; CV events: cardiovascular events

The risk scores for 15 distinct ADRs among the 2,387 members of the base cohort who had hyper-polypharmacy (concurrent use of ≥ 10 medications) were calculated based on the Cumulative Toxicity Tool in Polypharmacy Guidance Realistic Prescribing 2018, and the estimated cumulative burden of each ADR was visualized in the form of a heatmap (Fig. 1). The six ADRs with the highest cumulative burdens (individuals with a projected risk score of ≥ 5) were falls and fractures (*n* = 497, 20.8%), constipation (*n* = 275, 11.5%), CNS depression (*n* = 5.2%), and cardiovascular (CV) events (*n* = 101, 4.2%). The average projected risk scores were calculated for the 15 ADRs, and the highest risk scores were observed for falls and fractures (3.08), constipation (2.55), CV events (1.87), and CNS depression (1.70).

## Discussion

This large-scaled study was the first to investigate the burden of PIM use and risk of hospitalization among older adults living in RCHEs in Hong Kong. Moreover, this study adopted a more precise and pragmatic classification of PIMs than the 2023 AGS Beers criteria and exploited data on administered medications extracted from the SMMS^®^ database. Therefore, the descriptive findings of this study are reliable and accurately reflect the medications taken by the base cohort. It was found that more than a third (34.5%) of the base cohort took at least one PIM, particularly first-generation antihistamines and benzodiazepines. In addition, the use of a PIM was associated with 1.73 higher odds of hospitalization. Those who took at least two PIMs had 2.17-fold higher odds of hospitalization than those who did not take any PIM. These findings highlight the imminent need to develop local practical and sustainable measures to minimize the use of PIMs in older adults living in RCHEs. Moreover, there is a pressing need to engage pharmacists to review medication utilization among RCHE residents. Such a review may be more feasible if it is to lay focus on those who take the most common PIMs and who are at risk of the highest ADR burden from PIMs rather than include all those who take a PIM.

Within the study period, about 1 in 3 older adults of the base cohort took a PIM. Similarly, it has been found that 30.3–38.6% of community-dwelling older adults in Hong Kong have used a PIM [23, 28]. We acknowledge that some patient-related factors (e.g., age, sex, and comorbidity burden) are known unmodifiable risk factors for PIM use. For example, the majority of the current study population who took zolpidem and clonazepam received their prescriptions from a psychiatry hospital in Kowloon. This finding echoes with a study by Stuhec that reported high prevalence (25%) of potentially inappropriate antipsychotic use in nursing homes, and the need for collaborative strategies with pharmacists to ensure rational prescription of antipsychotics in older adults [29]. However, prescriber-related variables may also account for the high prevalence of PIM use by our cohort. Nevertheless, the prevalence of PIM use in our base cohort is lower than that reported by Zhang et al., i.e., 45.5%, in an investigation of PIM use among older adults visiting GOPCs in Hong Kong public healthcare system. These researchers outlined the underlying factors contributing to PIM use, including the highly subsidized services that led to a heavy patient load in the GOPCs and the absence of mandatory training on geriatric medicine for physicians practicing in the GOPCs [19]. We postulate that PIM use by the current study population is lower than that reported by Zhang et al. because our cohort mostly visited specialist outpatient clinics, which may have more well-trained physicians in geriatrics than GOPCs. Our findings reinforce the importance in increasing the competency of the physicians and RCHE staff through continuing professional education. Future work should be focused on collaborating with health administrators of the Hong Kong public healthcare system to adopt computerized decision-support tools to reduce potentially inappropriate prescriptions and the use of PIMs.

Consistent with previous findings [15, 30, 31], this study found that the use of PIMs in older adults at RCHEs was strongly associated with an increased risk of hospitalization, which increased with the number of PIMs used. This finding should be interpreted cautiously because PIM use and hospital admission data were both captured within the same study period. Although the analysis only counted hospital admissions after the start date of any PIM as a “case”, the cross-sectional design of this study meant that a causal relationship between PIM use and the risk of hospitalization could not be determined. We were also unable to differentiate between planned or unplanned admission due to limitations of the SMMS^®^. Nevertheless, based on previous studies, it is reasonable to postulate that drug–drug or drug–disease interactions and adverse events may be mechanisms by which PIMs can increase the risk of hospitalization [30, 32]. In addition, our exploratory analysis used the Cumulative Toxicity Tool in Polypharmacy Guidance Realistic Prescribing [27] to estimate the potential ADR risk associated each PIM. Although this estimation is hypothetical in nature and may not reflect the actual ADRs that the residents experienced, the analysis shows the highest ADR risk score for falls and fractures, which may logically be associated with a higher risk of hospitalization based on evidence from the literature [33, 34]. The strong association of PIMs with hospitalization and the “dose–response” increase in the risk of hospitalization observed in those of the study population who took at least two PIMs are further evidence of the detrimental effect of PIMs on older adults in RCHEs in Hong Kong.

Another finding of this study was that compared with those in the study population who took up to four medications, those who took from five to nine medications exhibited a lower risk of hospitalization, despite this number of medications traditionally being classified as polypharmacy. This finding is inconsistent with the findings in a review of previous studies [35] but could be explained by the concept of “appropriate polypharmacy ”[36]. This is a term for medicines prescribed according to best evidence to an individual who has complex conditions or multiple conditions and uses the medicines in an optimized fashion [37]. Therefore, we posit that in older adults with multiple comorbidities, the appropriateness and necessity of the medications prescribed are significant, in addition to the number of medications prescribed. However, this finding should be interpreted cautiously given that the concept of “appropriate polypharmacy” still requires validation and supporting evidence from the literature, and that the sample size of older adults who took five to nine medications is small. In summary, the clinical context in which multiple medications are prescribed should be considered to ensure that patients use only medications that are necessary for treating or managing their diseases and, if possible, that these medications are not PIMs [38].

Our findings reinforce the importance of regular medication review and reconciliation for residents in nursing homes. A recent review found that pharmacist-led services significantly reduced the mean number of falls among residents in RCHEs, although they did not uniformly reduce overall mortality and hospitalization rates [39]. However, most local RCHEs do not have in-house pharmacists who provide medication reviews for residents. To address this service gap, the recent Primary Healthcare Blueprint released by the Hong Kong government recommended that community geriatric assessment teams regularly visit RCHEs for older adults to provide medical and nursing care to frail residents [40]. We also found potentially inappropriate psychiatric medications prescribed to older adults in our study cohort. Several reports have demonstrated that including a clinical pharmacist in the interdisciplinary ward rounds and medication reconciliation reviews could significantly reduce potential drug-related problems in Slovenia [41, 42]; this has eventually led to the first national-level reimbursed medication review program in the primary care settings in Central Europe [11] In Germany, interdisciplinary pharmacist-led medication reviews have effectively led to increased recommendations in dose reductions and temporary or permanent discontinuation of PIMs in older adults with psychiatric conditions [43]. Similarly, countries such as Singapore and Australia have achieved cost savings and other positive outcomes by engaging community pharmacists from both public and private sectors to conduct regular medication reviews in RCHEs [44, 45]. The involvement of pharmacists in a multidisciplinary team is important for promoting the safe use of medications and implementing deprescribing practices in RCHEs.

A key strength of the current study is the use of an electronic medication management system to capture the medications administered to the study population. This approach allowed us to overcome several limitations reported in other studies using claims databases or dispensing records, such as a failure to account for drug adherence, incomplete medication data, and inaccurate coding [46, 47]. However, the current study also has several limitations. First, although the Beers Criteria is an internationally recognized criteria to categorize PIMs, the prevalence of PIM use might have been underestimated because as not all categories of the Beers criteria were assessed in the study. For example, PIMs related to indication, renal impairment, and drug-drug interactions were unaddressed because such information is not available in the SMMS^®^. To illustrate, a study by Kummer et al. has reported that more than a quarter (28%) of older adults living in RCHEs of Croatia were inappropriately prescribed with benzodiazepines higher than recommended geriatric doses and nearly half (48%) were prescribed concomitant interacting medications [48]. Therefore, the actual rate of PIM use may be even higher than what is reported in this study. Second, the use of the SMMS^®^ database restricted the assessment of medications to prescribed drugs, meaning that over-the-counter medications or supplements that the members of the base cohort might have been taking of their own accord were not assessed. Third, data on certain potential residual confounding factors, such as frailty status, disease severity, and disability, were not available to be included in our analyses. Furthermore, as medical diagnoses data in the Hospital Authority is currently not linked with the SMMS^®^, our manual approach to coding the diagnoses data may lead to errors in characterizing the comorbidities of the study population. Fourth, as this study leveraged on data collected from the Integrated Old Age Home Medication Management Program (a community service improvement project), there is no prior sample size calculation. However, we reckoned a study population consisting of 6,346 residents from 29 RCHEs may still be reasonably generalizable to the RCHE community in Hong Kong, as well as adequate to estimate the prevalence of PIM use and interpret the significant associations observed between PIM use and hospitalization. As the SMMS^®^ database does not contain information on resident mortality, we were unable to determine the survival status of a subgroup of residents (8.2%) who had been discharged before June 2024 due to death or other reasons. However, these residents comprised a small proportion of the base cohort, and thus it is unlikely that their discharge status significantly impacted the results.

## Conclusion

According to an assessment based on the latest version of the Beers criteria, there was a high burden of PIM use among older adults living in 29 RCHEs in Hong Kong during the study period. In addition, the use of PIMs by these older adults was associated with an increased risk of hospitalization, particularly for those who were taking at least two PIMs and had multiple comorbidities. Effective interventions should be implemented and strengthened in these RCHEs, particularly interventions that focus on educating physicians about PIMs and the use of computer-assisted technology in identifying PIMs. Future studies should examine the causes of PIMs in various settings and devise strategies that may ameliorate PIM-related healthcare burden.

## Electronic supplementary material

Below is the link to the electronic supplementary material.

Supplementary Material 1

## Data Availability

The data that support the findings of this study are available on request from the corresponding authors, YTC and SCC. The data are not publicly available due to information that could compromise the privacy of research participants.
