# Supplementary material for: Analysis of medication management system data to determine potentially inappropriate medication use and hospitalization among older adults living in residential care homes for the elderly population
Source: BMC Geriatr. 2025 May 6;25:314. doi: 10.1186/s12877-025-05989-4 (PMC12054255; doi:10.1186/s12877-025-05989-4)
Supplement: Supplementary file 1 — Supplementary Material 1 [file 12877_2025_5989_MOESM1_ESM.docx]

**Supplementary Figure 1.** Flowchart of participant inclusion into the cohort

Residents who were not administered any medications

(n = 1,660)

Residents who were younger than 65 years old

(n = 988)

Residents who were administered at least one medication from January 1 to December 31, 2023

(n = 4,686)

Residents who are aged 65 years or older and living in the participating RCHEs from January 1 to December 31, 2023

(n = 6,346)

Residents living in RCHEs which participated in the Integrated Old Age Home Medication Management Program from January 1 to December 31, 2023

(n = 7,334)

**Table S1.** Potentially inappropriate medications included in this study (adapted from the 2023 Beers Criteria)

| **Organ system** | **Therapeutic category** | **Medications** |
| --- | --- | --- |
| Antihistamines | First-generation antihistamines | Chlorpheniramine |
|  |  | Cyproheptadine |
|  |  | Dimenhydrinate |
|  |  | Diphenhydramine |
|  |  | Hydroxyzine |
|  |  | Promethazine |
| Cardiovascular | Antiplatelet | Dipyridamole (oral short-acting) |
|  | Calcium channel blockers | Nifedipine (immediate release) |
| Central nervous system | Antidepressants | Amitriptyline |
|  |  | Clomipramine |
|  |  | Doxepin >6 mg/day |
|  |  | Imipramine |
|  |  | Nortriptyline |
|  |  | Paroxetine |
|  | Antiparkinsonian agents | Benztropine (oral) |
|  |  | Trihexyphenidyl |
|  | Benzodiazepines | Alprazolam |
|  |  | Chlordiazepoxide |
|  |  | Clobazam |
|  |  | Clonazepam |
|  |  | Diazepam |
|  |  | Lorazepam |
|  |  | Midazolam |
|  |  | Triazolam |
|  | Nonbenzodiazepine | Zolpidem |
|  | Barbiturates | Phenobarbital (phenobarbitone) |
| Endocrine | Progestogens | Megestrol |
|  | Estrogens | Estrogens (oral, patch) |
|  | Sulfonylureas | Gliclazide |
|  |  | Glimepiride |
|  |  | Glipizide |
|  |  | Glibenclamide |
| Gastrointestinal | GI antispasmodics | Scopolamine (Hyoscine) |
| Pain medications | NSAIDs | Indomethacin |
|  |  | Ketorolac |
|  | Skeletal muscle relaxants | Chlorzoxazone |
|  |  | Orphenadrine |

**Table S2.** Prevalence of 32 groups of comorbidities in the base cohort (N=6,346)

| **Groups of comorbidities** | **Number of patients** | **Prevalence in %** |
| --- | --- | --- |
| **Neurological** |  |  |
| Dementia | 1,979 | 34.7 |
| Parkinson’s disease | 309 | 5.7 |
| Stroke | 1,798 | 31.3 |
| Epilepsy | 128 | 2.2 |
| **Cardiovascular** |  |  |
| Hypertension | 2,909 | 51.8 |
| Angina | 5 | 0.1 |
| Ischemic heart disease | 592 | 10.4 |
| Heart failure | 532 | 9.5 |
| Atrial fibrillation | 541 | 9.8 |
| **Cholesterol** |  |  |
| Cholesterol | 1,248 | 22.5 |
| **Endocrine** |  |  |
| Diabetes | 1,447 | 26.0 |
| Thyroid disease | 276 | 4.9 |
| **Respiratory** |  |  |
| COPD | 225 | 4.1 |
| Asthma | 95 | 1.7 |
| **Musculoskeletal** |  |  |
| Fractures | 1,195 | 21.1 |
| Osteoporosis | 412 | 7.3 |
| Arthritis | 874 | 15.6 |
| Gout | 395 | 7.0 |
| **Cancer** |  |  |
| Cancer | 622 | 11.5 |
| **Infection** |  |  |
| Pneumonia | 330 | 5.8 |
| Acute infection (Flu, cold, COVID) | 2,009 | 33.0 |
| UTI | 173 | 3.2 |
| **GI** |  |  |
| Peptic ulcer disease | 364 | 6.4 |
| Constipation | 100 | 1.8 |
| Diarrhea | 6 | 0.1 |
| **Skin** |  |  |
| Skin condition (Psoriasis, eczema) | 530 | 8.9 |
| **Eye** |  |  |
| Eye disease | 1,614 | 28.3 |
| **Renal** |  |  |
| Renal disease | 1,507 | 26.3 |
| **Liver** |  |  |
| Liver disease | 292 | 5.2 |
| **Psychiatric** |  |  |
| Psychiatric | 939 | 16.6 |
| **Blood and nutrition** |  |  |
| Anemia | 606 | 10.7 |
| Nutritional deficiency (vit D deficiency, vit B deficiency) | 298 | 5.1 |

**Table S3.** Medication usage patterns for the base cohort (N=6,346)

| **Medication** | **Number of patients** | **Prevalence of medication use in %** |
| --- | --- | --- |
| **Gastro-intestinal system** | 4381 | 69.0 |
| Bisacodyl | 2999 | 47.3 |
| Senna | 2811 | 44.3 |
| Lactulose | 2595 | 40.9 |
| Pantoprazole (Sodium Sesquihydrate) | 1308 | 20.6 |
| Famotidine | 1180 | 18.6 |
| Lansoprazole | 1056 | 16.6 |
| Esomeprazole (Magnesium Trihydrate) | 295 | 4.6 |
| Fleet (Or Equiv) | 206 | 3.2 |
| Glycerin (Adult) Rectal Suppository | 202 | 3.2 |
| Dimethylpolysiloxane | 169 | 2.7 |
| **Musculoskeletal and joint** | 1452 | 22.9 |
| Colchicine | 258 | 4.1 |
| Allopurinol | 210 | 3.3 |
| Celecoxib | 93 | 1.5 |
| Baclofen | 83 | 1.3 |
| Diclofenac Sodium | 56 | 0.9 |
| Ibuprofen | 35 | 0.6 |
| Febuxostat | 31 | 0.5 |
| Methotrexate | 20 | 0.3 |
| Mefenamic Acid | 16 | 0.3 |
| Etoricoxib | 15 | 0.2 |
| **Eye** | 1708 | 26.9 |
| Hypromellose | 1311 | 20.7 |
| Chloramphenicol | 163 | 2.6 |
| Systane Ultra (Or Equiv) | 75 | 1.2 |
| Xalacom (Or Equiv) | 70 | 1.1 |
| Timolol (Maleate) | 67 | 1.1 |
| Latanoprost | 65 | 1.0 |
| Simbrinza (Or Equiv) | 62 | 1.0 |
| Genteal (Or Equiv) | 56 | 0.9 |
| Brimonidine Tartrate | 55 | 0.9 |
| Maxitrol (Or Equiv) | 43 | 0.7 |
| **Ear, Nose, And Oropharynx** | 670 | 10.6 |
| Dequalinium Chloride | 229 | 3.6 |
| Chlorhexidine Gluconate | 207 | 3.3 |
| Povidone Iodine | 178 | 2.8 |
| Bonjela (Or Equiv) | 68 | 1.1 |
| Thymol Gargle Compound | 67 | 1.1 |
| Benzydamine HCl | 21 | 0.3 |
| Strepsils (Or Equiv) | 2 | 0.0 |
| **Skin** | 3145 | 49.6 |
| Heparinoid | 1063 | 16.8 |
| Aqueous cream | 1006 | 15.9 |
| Methyl Salicylate Compound | 956 | 15.1 |
| Clotrimazole | 880 | 13.9 |
| Fluocinolone Acetonide | 736 | 11.6 |
| Emulsifying | 648 | 10.2 |
| Crotamiton | 553 | 8.7 |
| Paraffin Soft White | 483 | 7.6 |
| Liquid Paraffin 50% + White Soft Paraffin 50% | 254 | 4.0 |
| Permethrin | 209 | 3.3 |
| **Immunological Products and Vaccines** | 305 | 4.8 |
| Influenza (2023/2024 Campaign) (Vaxigriptetra) | 194 | 3.1 |
| Darbepoetin Alfa (Nesp) | 58 | 0.9 |
| Denosumab (Prolia) | 51 | 0.8 |
| Covid-19(Comirnaty Orig./Omicron Ba.4-5) | 7 | 0.1 |
| Covid-19 (Coronavac) | 3 | 0.0 |
| Hepatitis B (Engerix B) | 1 | 0.0 |
| **Anaesthesia** | 383 | 6.0 |
| Lorazepam | 382 | 6.0 |
| Glycopyrrolate | 1 | 0.0 |
| **Cardiovascular System** | 4081 | 64.3 |
| Amlodipine (Besylate) | 2207 | 34.8 |
| Aspirin | 1763 | 27.8 |
| Simvastatin | 1368 | 21.6 |
| Losartan Potassium | 830 | 13.1 |
| Atorvastatin (Calcium) | 814 | 12.8 |
| Frusemide (Furosemide) | 799 | 12.6 |
| Metoprolol Tartrate | 615 | 9.7 |
| Lisinopril | 519 | 8.2 |
| Terazosin HCl | 392 | 6.2 |
| Glyceryl Trinitrate | 358 | 5.6 |
| **Respiratory System** | 2917 | 46.0 |
| Bromhexine HCl | 1336 | 21.1 |
| Chlorpheniramine Maleate | 1031 | 16.2 |
| Salbutamol (Sulphate) | 676 | 10.7 |
| Lysozyme Chloride | 577 | 9.1 |
| Loratadine | 420 | 6.6 |
| Ammonia and Ipecacuanha | 339 | 5.3 |
| Cetirizine HCl | 219 | 3.5 |
| Promethazine Compound | 199 | 3.1 |
| Diphenhydramine Compound | 169 | 2.7 |
| Acetylcysteine | 168 | 2.6 |
| **Central Nervous System** | 4350 | 68.5 |
| Memantine HCl | 640 | 10.1 |
| Tramadol HCl | 582 | 9.2 |
| Zopiclone | 572 | 9.0 |
| Quetiapine (Fumarate) | 520 | 8.2 |
| Trazodone HCl | 473 | 7.5 |
| Mirtazapine | 333 | 5.2 |
| Metoclopramide HCl | 272 | 4.3 |
| Sertraline (HCl) | 263 | 4.1 |
| Betahistine Mesylate | 248 | 3.9 |
| Donepezil HCl | 246 | 3.9 |
| Phenobarbital (phenobarbitone) | 13 | 0.2 |
| **Infections** | 2573 | 40.5 |
| Augmentin (Or Equiv) | 1730 | 27.3 |
| Molnupiravir | 600 | 9.5 |
| Levofloxacin | 404 | 6.4 |
| Paxlovid (Or Equiv) | 269 | 4.2 |
| Fusidic Acid | 238 | 3.8 |
| Oseltamivir (Phosphate) | 161 | 2.5 |
| Ciprofloxacin (HCl) | 145 | 2.3 |
| Paxlovid (Or Equiv) (Renal Impairment) | 132 | 2.1 |
| Entecavir | 111 | 1.7 |
| Doxycycline Hyclate | 108 | 1.7 |
| **Endocrine System** | 1878 | 29.6 |
| Metformin HCl | 768 | 12.1 |
| Gliclazide | 280 | 4.4 |
| Thyroxine Sodium | 240 | 3.8 |
| Prednisolone | 234 | 3.7 |
| Linagliptin | 212 | 3.3 |
| Urea | 176 | 2.8 |
| Hydrocortisone | 147 | 2.3 |
| Alendronate Sodium | 141 | 2.2 |
| Finasteride | 125 | 2.0 |
| Vildagliptin | 64 | 1.0 |
| **Obstetrics, Gynaecology, And Urinary-Tract Disorders** | 570 | 9.0 |
| Tamsulosin HCl | 244 | 3.8 |
| Prazosin (HCl) | 126 | 2.0 |
| Phenazopyridine HCl | 57 | 0.9 |
| Alfuzosin HCl | 53 | 0.8 |
| Distigmine Bromide | 50 | 0.8 |
| Solifenacin Succinate | 23 | 0.4 |
| Amitriptyline HCl | 22 | 0.3 |
| Oxybutynin HCl | 19 | 0.3 |
| Duodart (Or Equiv) | 13 | 0.2 |
| Tolterodine Tartrate | 2 | 0.0 |
| **Malignant Disease and Immunosuppression** | 145 | 2.3 |
| Megestrol Acetate | 44 | 0.7 |
| Letrozole | 29 | 0.5 |
| Hydroxyurea (Hydroxycarbamide) | 10 | 0.2 |
| Osimertinib (Mesylate) | 9 | 0.1 |
| Capecitabine | 7 | 0.1 |
| Exemestane | 7 | 0.1 |
| Tamoxifen (Citrate) | 6 | 0.1 |
| Bicalutamide | 6 | 0.1 |
| Mycophenolate Mofetil (Micocept) | 5 | 0.1 |
| Tacrolimus | 4 | 0.1 |
| **Nutrition and Blood** | 3294 | 51.9 |
| Calcium (Carbonate) + Vitamin D | 1054 | 16.6 |
| Potassium Chloride | 900 | 14.2 |
| Cyanocobalamin (Vit B12) | 649 | 10.2 |
| Zinc | 501 | 7.9 |
| Folic Acid | 447 | 7.0 |
| Ferrous Sulphate | 426 | 6.7 |
| Sodium Chloride | 415 | 6.5 |
| Mecobalamin | 324 | 5.1 |
| Thiamine HCl (Vit B1) | 300 | 4.7 |
| Calcium Carbonate | 290 | 4.6 |

**Table S4.** 12-month prevalence of potentially inappropriate medications use in the base cohort (N=6,346)

| **Medication** | **Number of patients** | **Prevalence of PIM use in %** | **Class of medications** |
| --- | --- | --- | --- |
| Chlorpheniramine | 1,031 | 16.25 | First-generation antihistamines |
| Lorazepam | 382 | 6.02 | Benzodiazepines |
| Promethazine | 288 | 4.54 | First-generation antihistamines |
| Gliclazide | 280 | 4.41 | Sulfonylureas |
| Clonazepam | 175 | 2.76 | Benzodiazepines |
| Diphenhydramine | 171 | 2.69 | First-generation antihistamines |
| Trihexyphenidyl | 125 | 1.97 | Antiparkinsonian agents |
| Hydroxyzine | 109 | 1.72 | First-generation antihistamines |
| Zolpidem | 105 | 1.65 | Nonbenzodiazepine |
| Scopolamine (Hyoscine) | 102 | 1.61 | GI antispasmodics |
| Cyproheptadine | 81 | 1.28 | First-generation antihistamines |
| Glipizide | 54 | 0.85 | Sulfonylureas |
| Dimenhydrinate | 50 | 0.79 | First-generation antihistamines |
| Diazepam | 48 | 0.76 | Benzodiazepines |
| Alprazolam | 44 | 0.69 | Benzodiazepines |
| Megestrol | 44 | 0.69 | Progestogens |
| Dipyridamole (oral short-acting) | 27 | 0.43 | Antiplatelet |
| Glimepiride | 24 | 0.38 | Sulfonylureas |
| Amitriptyline | 22 | 0.35 | Antidepressants |
| Paroxetine | 15 | 0.24 | Antidepressants |
| Phenobarbital | 13 | 0.2 | Barbiturates |
| Nortriptyline | 7 | 0.11 | Antidepressants |
| Indomethacin | 4 | 0.06 | NSAIDs |
| Imipramine | 4 | 0.06 | Antidepressants |

**Table S5.** Characteristics of hospitalized versus non-hospitalized residents who were administered at least 1 medication in 2023

| Variables | Total (n=4,686)  n (%) | Non-hospitalized residents (n=2,123)  n (%) | Hospitalized residents (n=2,563)  n (%) | *P* |
| --- | --- | --- | --- | --- |
| PIM use in 2023 |  |  |  | **<0.0001** |
| No | 2496 (53.3) | 1293 (60.9) | 1203 (46.9) |  |
| Yes | 2190 (46.7) | 830 (39.1) | 1360 (53.1) |  |
| Sex |  |  |  | **0.037** |
| Female | 2974 (63.5) | 1382 (65.1) | 1592 (62.1) |  |
| Male | 1712 (36.5) | 741 (34.9) | 971 (37.9) |  |
| Age |  |  |  | **0.0005** |
| 65–74 | 582 (12.4) | 276 (13.0) | 306 (11.9) |  |
| 75–84 | 1169 (25.0) | 582 (27.4) | 587 (22.9) |  |
| 85–94 | 2167 (46.2) | 949 (44.7) | 1218 (47.5) |  |
| ≥95 | 768 (16.4) | 316 (14.9) | 452 (17.6) |  |
| No. of concurrent medications |  |  |  | **<0.0001** |
| 0-4 | 1755 (37.5) | 798 (37.6) | 957 (37.3) |  |
| 5-9 | 765 (16.3) | 414 (19.5) | 351 (13.7) |  |
| ≥10 | 2166 (46.2) | 911 (42.9) | 1255 (49.0) |  |
| No. of PIMs in 2023 |  |  |  | **<0.0001** |
| 0 | 2496 (53.3) | 1293 (60.9) | 1203 (46.9) |  |
| 1 | 1426 (30.4) | 581 (27.4) | 845 (33.0) |  |
| ≥1 | 764 (16.3) | 249 (11.7) | 515 (20.1) |  |
| No. of comorbidities |  |  |  | **<0.0001** |
| 0-2 | 1376 (29.4) | 711 (33.5) | 665 (26.0) |  |
| 3-5 | 1217 (26.0) | 574 (27.0) | 643 (25.1) |  |
| 6-8 | 1129 (24.1) | 489 (23.0) | 640 (25.0) |  |
| ≥9 | 964 (13.1) | 240 (11.3) | 375 (14.6) |  |

PIM: potentially inappropriate medication.
